# Supplementary material for: Resolving the full spectrum of human genome variation using Linked-Reads
Source: Genome Res. 2019 Apr;29(4):635–45. doi: 10.1101/gr.234443.118 (PMC6442396; doi:10.1101/gr.234443.118)
Supplement: Supplemental Material [file supp_gr.234443.118_Supplemental_longranger-2.2.zip › longranger-2.2.tar.bz2!/lr2.2-public/lib/stan/cmdstan/stan/doc/api/html/fwd_2functions_2fabs_8hpp_source.html]

Stan: src/stan/agrad/fwd/functions/fabs.hpp Source File


|  |  |
| --- | --- |
|  | Stan  2.5.0  probability, sampling & optimization |


- Main Page
- Related Pages
- Namespaces
- Classes
- Files

- File List
- File Members

All Classes Namespaces Files Functions Variables Typedefs Enumerations Enumerator Friends Macros Pages

- src
- stan
- agrad
- fwd
- functions

fabs.hpp

Go to the documentation of this file.

1 #ifndef STAN\_\_AGRAD\_\_FWD\_\_FUNCTIONS\_\_FABS\_HPP

2 #define STAN\_\_AGRAD\_\_FWD\_\_FUNCTIONS\_\_FABS\_HPP

3

4 #include <stan/agrad/fwd/fvar.hpp>

5 #include <stan/meta/traits.hpp>

6 #include <stan/math/constants.hpp>

7 #include <stan/math/functions/value\_of.hpp>

8 #include <math.h>

9 #include <stan/meta/likely.hpp>

10

11 namespace stan {

12

13  namespace agrad {

14

15  template<typename T>

16  inline

17  fvar<T>

18  fabs(const fvar<T>& x) {

19  using stan::math::NOT\_A\_NUMBER;

20  using ::fabs;

21  using stan::math::value\_of;

22

23  if (unlikely(boost::math::isnan(value\_of(x.val\_))))

24  return fvar<T>(fabs(x.val\_),stan::math::NOT\_A\_NUMBER);

25  else if (x.val\_ > 0.0)

26  return x;

27  else if (x.val\_ < 0.0)

28  return fvar<T>(-x.val\_, -x.d\_);

29  else

30  return fvar<T>(0, 0);

31  }

32  }

33 }

34 #endif

boost::math::isnan

bool isnan(const stan::agrad::var &v)

Checks if the given number is NaN.

**Definition:** boost\_fpclassify.hpp:69

stan::agrad::fabs

fvar< T > fabs(const fvar< T > &x)

**Definition:** fabs.hpp:18

stan::math::NOT\_A\_NUMBER

const double NOT\_A\_NUMBER

(Quiet) not-a-number value.

**Definition:** constants.hpp:53

stan::agrad::fabs

var fabs(const var &a)

Return the absolute value of the variable (cmath).

**Definition:** fabs.hpp:53

stan::agrad::fvar

**Definition:** fvar.hpp:13

stan::agrad::value\_of

T value\_of(const fvar< T > &v)

Return the value of the specified variable.

**Definition:** value\_of.hpp:16

traits.hpp

stan::math::value\_of

double value\_of(const T x)

Return the value of the specified scalar argument converted to a double value.

**Definition:** value\_of.hpp:24

unlikely

#define unlikely(x)

**Definition:** likely.hpp:9

fvar.hpp

likely.hpp

value\_of.hpp

stan::agrad::fvar::d\_

T d\_

**Definition:** fvar.hpp:16

constants.hpp

stan::agrad::fvar::val\_

T val\_

**Definition:** fvar.hpp:15


---

[ Stan Home Page ]

*© 2011–2014,
Stan Development Team.*
